# Stent placement as rescue treatment in acute basilar artery occlusion

**DOI:** 10.1007/s00415-026-13922-x

**Published:** 2026-06-08

**Authors:** Susanna Sammali, Danilo Caimano, Francesco Capasso, Leonardo Renieri, Anna Poggesi, Cristina Sarti, Francesca Pescini, Maria Lamassa, Benedetta Piccardi, Vanessa Palumbo, Mascia Nesi, Nicola Limbucci, Antonio Laiso, Giorgio Busto, Enrico Fainardi, Patrizia Nencini, Francesco Arba

**Affiliations:** 1 University of Florence, Florence, Italy; 2 Neurology Unit, Cerebrovascular and Neurodegenerative Disease Area of the Department of Medical Specialties, Central Tuscany Local Health Authority, Florence, Italy; 3 Neurovascular Interventional Unit, Careggi University Hospital, Florence, Italy; 4 Stroke Unit, Careggi University Hospital, Florence, Italy; 5 Department of Radiology, Neuroradiology Unit, Careggi University Hospital, Florence, Italy; 6 Department of Experimental and Clinical Biomedical Sciences, Neuroradiology Unit, University of Florence, Florence, Italy

**Keywords:** Ischemic stroke, Basilar artery, Endovascular treatment, Stenting, Outcome

## Abstract

**Introduction:**

We investigated the etiology, safety and efficacy of endovascular treatment with and without stent placement as rescue treatment in patients with acute ischemic stroke due to basilar artery occlusion (BAO).

**Methods:**

A Single-center retrospective observational study in consecutive patients with BAO treated with endovascular procedures, divided into stent and non-stent groups. Outcomes of interest were: stroke etiology; recanalization of the basilar artery defined as mTICI ≥ 2b; any intracranial hemorrhage; poor functional outcome (mRS 4–6) at 3 months. Independent associations were investigated with multivariable logistic and ordinal regression analyses adjusted for age, sex, NIHSS, rt-PA use and time-to-groin.

**Results:**

The analysis included 121 patients with a mean age of 70 (± 14) years; 48 (40%) were female. The median NIHSS score was 14 (IQR 6–36). Stent placement was performed in 28 patients (23%), mainly in those with atherosclerosis and artery dissection. Recanalization occurred in 110 (91%) patients, 79% in the stent group patients and 94% in the non-stent group. Ordinal analysis demonstrated higher risk of worse outcome in the stent group (OR = 3.73; 95%CI = 1.46–9.57); however, all patients without vessel recanalization had poor functional outcome. Periprocedural complications, particularly vessel perforation (14% vs 2%, *p* < 0.001), were more frequent in the stent group, whereas intracranial hemorrhage was similar between groups (7% vs 2%, *p* = 0.200).

**Conclusions:**

Stent placement was more frequent in patients with stroke due to atherosclerosis or artery dissection and was associated with higher rates of procedural complications and poor functional outcome. However, stenting represents an acceptable option for rescue therapy in BAO due to the high burden of disease in patients who did not achieve vessel recanalization.

**Supplementary Information:**

The online version contains supplementary material available at 10.1007/s00415-026-13922-x.

## Introduction

Basilar artery occlusion (BAO) is a rare presentation of acute cerebrovascular disease, accounting for 1% of all ischemic strokes [1]. Clinical manifestations of BAO are highly variable, and patients may show a wide range of clinical presentations, ranging from typical focal symptoms with sudden onset and atypical vascular syndromes to rapidly progressive brainstem dysfunction or loss of consciousness, leading to misdiagnosis and delay in treatment [2]. Moreover, BAO has a frequently unfavorable prognosis, with mortality ranging from around 30% to 40% in EVT-treated patients and up to 80% of patients having functional disability [3–7].

Recanalization of the occluded vessel is a powerful prognostic factor for a favorable outcome and is the main target of EVT. Permanent stent placement has been suggested as a rescue therapy for achieving recanalization in refractory occlusions in anterior circulation stroke [8]. However, data regarding safety and efficacy of rescue therapy with stent placement in the basilar artery are scarce [4, 9–11]. The Basilar Artery Occlusion Chinese Endovascular (BAOCHE) and the Endovascular Treatment for Acute Basilar-Artery Occlusion (ATTENTION) trials, mainly on Asiatic population, demonstrated the superiority of EVT plus best medical care over best medical care alone for patients with BAO and moderate-severe stroke, with around half of the included patients treated with intracranial angioplasty or stenting as rescue therapy [5, 12, 13]. Stenting of basilar artery occlusion represents a marker of disease and procedural complexity and is performed after failure of mechanical thrombectomy. Stenting can complicate with dissection, distal embolization, vessel rupture, and occlusion of brainstem perforating arteries. Also, the use of periprocedural or post-procedural antiplatelet medication might increase the risk of intracranial bleeding [14, 15]. Finally, there are scarce data regarding the etiology of stroke in such patients.

In this study on patients with BAO treated with endovascular therapy, we compared periprocedural complications and clinical outcome, and analyzed differences in stroke etiology in those without and those with stent placement.

## Materials and methods

### Study design

This was a hospital-based, retrospective, single-center, observational study. We extracted patient data from the clinical charts of the Stroke Unit at a comprehensive University Hospital. We included consecutive patients with isolated BAO treated with EVT between April 2015 and December 2023. We followed the Strengthening the Reporting of Observational Studies in Epidemiology (STROBE) recommendations for observational studies. [16]. The study was approved by the local Ethics Committee (N. 13068/OSS). Where possible, patients provided informed consent within 48 h or at the follow-up visit. This research was conducted ethically in accordance with the World Medical Association Declaration of Helsinki. The data supporting the findings of this study are available from the corresponding author upon reasonable request.

### Inclusion criteria

All included patients underwent computed tomography (CT) and computed tomography angiography (CTA) before endovascular treatment. We included patients with: (1) an acute ischemic stroke, defined as a sudden onset of focal neurological deficit attributable to vascular dysfunction; (2) evidence of basilar artery occlusion on computed tomography angiography (CTA) and confirmed with digital subtraction angiography (DSA) during endovascular treatment; (3) time from symptom onset to groin puncture of up to 6 h or, if more than six hours, evidence of posterior circulation Alberta Stroke Program Early CT score of more than 5, assessed with magnetic resonance; and (4) endovascular treatment (thrombectomy/thromboaspiration, stent retriever, and/or angioplasty vs stent placement) with or without previous rt-PA (recombinant tissue plasminogen activator) administration. Diagnosis was performed by clinical examination and evidence of BAO on CTA before endovascular treatment.

We excluded patients with: (1) recurrent ischemic stroke, (2) anterior circulation ischemic stroke, (3) simultaneous occlusion of anterior and posterior circulation arteries, or (4) evidence of hemorrhage on imaging. We also excluded patients with missing clinical data and those who did not undergo EVT for the following reasons: non-occlusive stenosis, unfavorable risk–benefit ratio in BAO with mild symptoms, no evidence of BA occlusion, clinical improvement without treatment, and inability to reach the occlusion site (technical failure of endovascular treatment).

### Clinical data

Baseline demographic and clinical data were extracted from clinical charts for all study participants. Baseline demographic factors included age, sex, cardiovascular risk factors (habitual or current smoking, hypertension, hypercholesterolemia), and pre-stroke functional status, expressed with the modified Rankin Scale (mRS). The presence of previous stroke or transient ischemic attack (TIA), ischemic heart disease, diabetes mellitus, and atrial fibrillation was recorded. Clinical data at admission and during hospitalization were collected. Stroke severity was measured with the National Institute of Health Stroke Scale (NIHSS) and/or with the Glasgow Coma Scale (GCS) by a stroke neurologist. Laboratory data were recorded. All patients underwent a 12-lead ECG and echocardiography. Stroke etiology was classified using the TOAST system into large artery atherosclerosis, cardioembolism, stroke of other determined cause (e.g., dissection), and stroke of undetermined cause. For the assessment of the stroke etiology, two neurologists (SS and FA) reviewed all clinical and outpatients’ records, and a neurointerventionalist (LR) reviewed all intraprocedural angiograms for basilar or vertebral artery stenosis or dissection. The final decision was made by consensus.

### Endovascular treatment data

All patients underwent endovascular treatment. The choice of the technique and type of treatment was at the discretion of the interventional neuroradiologist. Endovascular treatments were classified as follows: thrombectomy and/or thrombus aspiration as a first-line treatment, and angioplasty with stent placement as a rescue treatment. At our institution, angioplasty/stent placement was considered only after failure of three or more thrombectomy and/or thrombus aspiration maneuvers as a rescue approach in refractory occlusions. We therefore analyzed two separate groups: patients treated without stent placement (thrombectomy/thromboaspiration) and patients treated with stent placement (including those receiving percutaneous transluminal angioplasty, PTA). Technical difficulties in performing endovascular maneuvers, repeated passages through the vessels, occlusion due to the presence of an atherosclerotic plaque or vessel dissection, or the tendency to vessel reocclusion were factors that influenced the choice towards a rescue therapy with stenting. Final recanalization of the basilar artery was defined with the modified treatment in cerebral infarction score (mTICI) [17]. In case of stent placement, intravenous infusion of tirofiban or cangrelor was started as soon as demonstration of vessel recanalization was achieved. Intravenous infusion was continued for 6–12 h; therefore we shifted to double antiplatelet therapy (aspirin 100 mg plus clopidogrel 75 mg or ticagrelor 90 mg bid in case of non-responders to clopidogrel) after exclusion of intracerebral hemorrhage and demonstration of stent patency with Anglo-CT scan.

### Outcomes

Our primary outcome of interest was poor functional outcome at three months after the index stroke defined as a mRS 4 to 6 at 90 days after the index stroke. Other efficacy outcomes of interest were: (1) a shift on the ordinal mRS; (2) early neurological improvement, defined as an improvement of four or more points at NIHSS within the first 24 h after the endovascular procedure. Good recanalization was defined as a score of at least 2b, 2c or 3 on mTICI. Safety outcomes of interest were: (1) any hemorrhagic transformation of ischemic stroke within 24 h, defined with the European Cooperative Acute Stroke Study II (ECASS II) classification; (2) periprocedural complications (distal embolization or perforation of vessels); (3) orotracheal intubation (OTI) within 24 h after endovascular treatment; and (4) death at three months. We also described the etiology of the index stroke in the non-stent and stent groups.

### Statistical analysis

We described general characteristics of the population with summary statistics, and used analysis of variance, Mann–Whitney U test and Pearson χ^2^ as appropriate, to test differences between the two groups (stent and non-stent). We therefore evaluated univariate associations between groups and study outcomes using logistic and ordinal regression for binary and ordinal outcomes, respectively. We retained in multivariate analysis explanatory variables with *p* < 0.1 in the univariate analysis adjusting for age, sex, NIHSS, rt-PA and time-to-groin. We considered statistically significant a P value < 0.05. Statistical analysis was carried out using SPSS for Mac (V.29.0.2.0; SPSS, IBM Corp, Armonk, New York, USA).

## Results

### Characteristics of population

Data on 3974 patients admitted to the Stroke Unit Department from April 2015 to December 2023 were screened. A total of 166 (4%) had a diagnosis of isolated BAO: among these remaining patients, we excluded those who did not undergo endovascular treatment or whose clinical data were unavailable (poor imaging quality, incomplete clinical or outcome data). The final population consisted of 121 (73% of the whole BAO, 92% of all BAO treated with endovascular procedures) patients (see Supplementary Materials). Baseline characteristics of patients are summarized in Table 1.

**Table 1:** Baseline characteristics of study population

	Total *N* = 121	Stent *N* = 28	Non-stent *N* = 93	*p* value
Age, mean (± SD)	70 ± 14	66 ± 13	72 ± 14	**0.042**
Sex, female	48 (40)	7 (25)	41 (44)	**0.070**
NIHSS at admission, median (IQR)	14 (6–36)	16 (4–36)	12 (6–33)	0.513
GCS at onset, mean (± SD)	14 (7–15)	14 (7–15)	14 (7–15)	0.916
SBP at admission, mmHg, mean (± SD)	148 ± 25	150 ± 25	147 ± 26	0.567
DBP at admission, mmHg, mean (± SD)	74 ± 15	75 ± 14	74 ± 15	0.663
Glucose at admission, mg/dl, mean (± SD)	148 ± 61	164 ± 55	144 ± 63	0.134
Pre-stroke mRS 0–1	100 (83)	21 (91)	79 (91)	0.941
Previous TIA/stroke	20 (17)	4 (14)	16 (17)	0.715
Current tobacco use	15 (12)	3 (11)	12 (13)	0.758
DM	26 (21)	9 (32)	17 (18)	0.117
AF	17 (14)	2 (7)	15 (16)	0.230
Hypertension	79 (65)	20 (71)	59 (63)	0.436
Dyslipidemia	28 (23)	7 (25)	21 (23)	0.790
rt-PA	43 (36)	8 (29)	35 (38)	0.380
Site of occlusion				** < 0.001**
*Proximal*	17 (14)	7 (25)	10 (11)	
*Middle*	46 (38)	18 (64)	28 (30)	
*Distal*	58 (48)	3 (11)	55 (59)	
Time to groin, minutes, median (IQR)	281 (185–413)	373 (181–548)	270 (188–373)	**0.096**
Length of treatment, minutes, median (IQR)	80 (44–120)	90 (63–129)	75 (40–120)	**0.072**
Etiology:				** < 0.001**
*CE*	37 (30)	1 (4)	36 (39)	
*LAD*	44 (36)	20 (71)	24 (26)	
*Other (e.g., dissection)*	14 (12)	6 (21)	8 (8)	
*Unknown*	26 (22)	1 (4)	25 (27)	

Values are numbers (%) unless otherwise specified

*AF* atrial fibrillation, *CE* cardio-embolism, *DBP* diastolic blood pressure, *DM* diabetes mellitus, GCS Glasgow Coma Scale, *IQR* interquartile range, *LAD* large artery disease, *mRS* modified Rankin Scale, *NIHSS* National Institute Health Stroke Scale, *SBP* systolic blood pressure, *rt-PA* recombinant tissue plasminogen activator, *SD* standard deviation, *TIA* transient ischemic attack

Mean age was 70 (± 14) years, 48 (40%) were female and 43 (36%) received bridging therapy with rt-PA. 100 (83%) had a pre-stroke mRS of 0 or 1. Median (IQR) NIHSS at admission was 14 (6–36) and median (IQR) GCS at onset was 14 (7–15). Median (IQR) time-to-groin was 281 (185–413) minutes. Of 121 patients who underwent EVT, 28 (23%) were treated with stent placement, while 93 (77%) received treatment with thrombectomy/thromboaspiration, stent retriever and/or cerebral angioplasty (non-stent group). Overall, patients in the stent group were younger (66 vs 72 years; p = 0.042), less frequently female (25% vs 44%; *p* = 0.070) and experienced vessel occlusion more frequently in the proximal (25% vs 11%; *p* < 0.001) and middle (64% vs 30%; *p* < 0.001) segments of the basilar artery, while the non-stent group had more frequently distal occlusions (59% vs 11%; *p* < 0.001). Compared with patients treated without stent placement, the majority of patients treated with stent placement had large artery atherosclerosis with basilar artery stenosis (71% vs 26%, p < 0.001), followed by artery dissection (21% vs 8%, *p* < 0.001), whereas cardioembolic and unknown origins were rare. Time-to-groin (373 vs 270 min; *p* = 0.096) and time from onset to the end of the treatment (460 vs 360 min; *p* = 0.045) were higher in the stent group. There were no differences concerning other stroke baseline risk factors between the groups.

### Functional outcomes

**Fig. 1:**
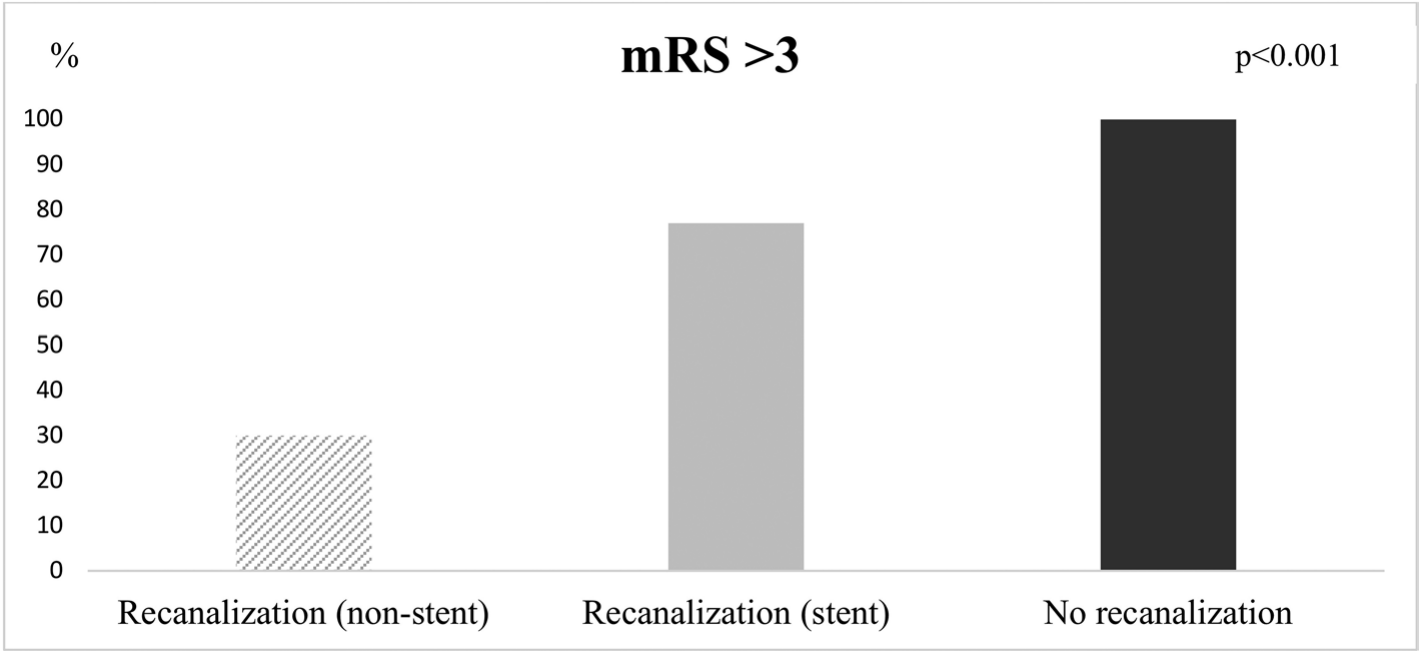
Poor functional outcome at 3 months stratified for recanalization (without stent and with stent) and for no recanalization

**Fig. 2:**
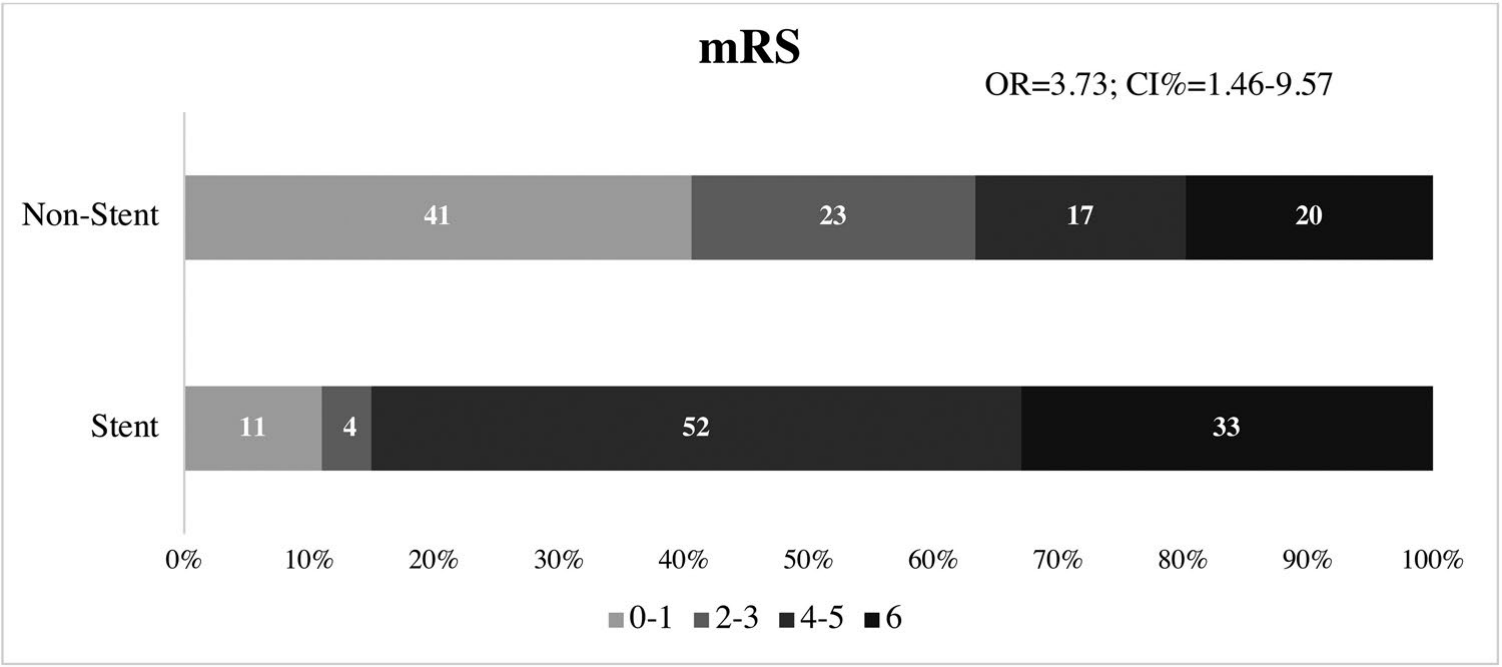
Distribution of mRS at 3 months after index stroke. OR is from ordinal analysis adjusted for age, sex, rt-PA treatment, National Institutes of Health Stroke Scale score and onset-to-groin puncture time

At 3 months, a total of 26 (22%) patients had died, and 54 (45%) had poor functional outcome. All (11/11) patients without vessel recanalization had poor functional outcome at three months compared with 77% (17/22) of patients with vessel recanalization with stent and 30% (26/88) with vessel recanalization with endovascular thrombectomy, *p* < 0.001 (Fig. 1). This association was confirmed in multivariable logistic regression analysis (OR = 16.30; 95%CI = 3.94–67.52). In ordinal shift analysis, after adjustment for confounders, we found a three-fold risk shift increase towards worse functional outcome at three months (common OR = 3.73; 95%CI = 1.46–9.57) in patients with stent placement (Fig. 2). The rate of neurological improvement within 24 h was lower in the stent group (7% vs 38%; *p* = 0.002) (Table 2). The association was confirmed after adjustment for confounders (OR = 0.15; 95%CI = 0.032–0.72).

**Table 2:** Recanalization, functional and safety outcomes stratified for treatment group (non-stent vs stent)

	Total *N* = 121	Stent *N* = 28	Non-stent *N* = 93	*p* value
Poor functional outcome (mRS > 3)	54 (45)	23 (82)	31 (33)	** < 0.001**
Recanalization, mTICI 2b/2c/3	110 (91)	22 (79)	88 (94)	**0.022**
Periprocedural complications:				**0.034**
*None*	110 (91)	23 (82)	87 (94)	
*Distal embolization/Fragmentation*	3 (3)	1 (4)	2 (2)	
*Perforation*	6 (5)	4 (14)	2 (2)	
Any ICH at 24 h	4 (3)	2 (7)	2 (2)	0.200
OTI at 24 h	54 (47)	20 (71)	34 (37)	**0.002**
Early Neurological improvement	37 (31)	2 (7)	35 (38)	**0.002**
Death at 3 months	26 (22)	9 (33)	17 (20)	0.162

Values are number (%). *ICH* intracranial hemorrhage, *mTICI* modified Treatment in Cerebral Infarction Score, *mRS* modified Rankin Scale*, OTI* orotracheal intubation

### Post-procedural and safety outcomes

Good recanalization was obtained in 110 (91%) of patients, with a higher frequency in non-stent EVT treatment (94% vs 79% respectively; *p* = 0.022). Periprocedural complications were more frequent in the stent group: embolization was present in 4% (vs 2%) and perforation in 14% (vs 2%) of patients. There were no differences in any ICH between the two groups; however, the chance of being intubated after treatment was almost doubled in the stent group (71% vs 37%; *p* = 0.002) (Table 2).

## Discussion

In patients with BAO treated with EVT we investigated safety and efficacy outcomes of patients who underwent stent placement as rescue therapy compared to those who received standard EVT. We found that despite high recanalization rates in both groups, early neurological improvement was less frequent and poor functional outcomes were more frequent in the stenting group compared to the non-stenting group. Periprocedural complications were more frequent in the stenting group, whereas intracranial hemorrhage and death were similar in both groups. The different etiology of the stroke we observed may explain the failure of endovascular maneuvers and favor the decision of stent placement as rescue therapy.

BAO, although rare, represents one of the most disabling subtypes of stroke and survivors are burdened by high rates of functional disability [18]. Some studies estimated that almost 60% of BAO survivors have moderate to severe disability, highlighting the serious long-term impact of this condition [6, 19]. Given that the recanalization of the occluded vessel is one of the most powerful predictors of a favorable outcome [20], stent placement as rescue therapy appears justified in case of failure of mechanical thrombectomy and/or thrombus aspiration in order to achieve recanalization of the basilar artery in BAO. Our results seem to confirm this hypothesis, showing a recanalization rate of around 90%, in keeping with recent trials [5, 12], whereas all patients who did not achieve recanalization had poor functional outcome at 3 months. However, clinical trials did not specifically address safety and efficacy of stent placement in BAO, despite it being used in more than one third of patients enrolled [5, 12]. The recent European Stroke Organization (ESO) guidelines suggested stent placement as a rescue therapy after failure of endovascular thrombectomy, but the few available recommendations have been formulated by expert consensus, suggesting that conclusive evidence is lacking [17, 21].

We observed that the patients in the stent group had mainly atherosclerosis as the underlying cause of the vessel occlusion. Our results are in keeping with a recent smaller study which reported a higher frequency of large artery atherosclerosis in patients who had refractory endovascular treatment and required stent placement [22]. However, available data are mainly based on an Asiatic population, with a different underlying stroke etiology profile compared to other ethnicities. In fact, in the aforementioned trials, atherosclerosis was the cause of vessel occlusion in almost half of patients, and a stent was placed in more than one third of the whole population assigned to the endovascular treatment arm [5, 12, 23]. In our cohort this figure was slightly lower, since we found that atherosclerosis was the cause of stroke in around a third of the whole population, and around a fourth of patients were treated with stent placement. Such data suggest stroke caused by atherosclerosis in the basilar artery is associated with higher chances of stent placement due to refractory occlusion. The peculiar anatomy of the vascularization territory of the basilar artery may represent another critical issue hindering stent placement and resulting in the occlusion of perforating arteries, leading to relevant neurological deficits due to truncal ischemia [24]. Although the presence of large vessel atherosclerosis may be suspected in some cases, identification of stroke etiology during endovascular treatment is not straightforward, and the choice of stent placement should be considered only in case of failure of endovascular thrombectomy/thrombus aspiration and not as a first-line treatment choice.

However, we observed that acute BAO could benefit from stenting, with vessel recanalization obtained in more than two-thirds of cases, without a significant increase of hemorrhagic transformation, despite an increase of periprocedural complications. Our results showed that in the overall population 80% of patients treated with endovascular therapy achieved recanalization of the basilar artery, whereas this figure was lower in patients with stent placement. The longer intraprocedural treatment time in patients with stent placement could be due to technical difficulties of revascularization (i.e., refractory occlusion, anatomical issues) and may partly explain the consequent higher rate of periprocedural complications in the stenting group, including vessel perforation and hemorrhagic complications. Consequently, the lower rate of early neurological improvement and the worse functional outcome at 3 months reflected not only the lower recanalization rate but also the periprocedural complications and the longer time-to-groin in patients who underwent stenting. However, given that artery stenting is a rescue therapy, our data indirectly suggest that stenting is necessary to achieve recanalization in around a fifth of patients with BAO. Considering the high burden of disability of BAO and the poor functional outcome that we found in the few patients who did not achieve recanalization, stent placement appears as a reasonable trade-off between risks and benefits and an acceptable choice as a rescue therapy.

Our study has limitations. First, the limited sample size suggests caution in the interpretation of the data, and the retrospective and non-randomized nature of our cohort did not allow generalization of the results which are hypothesis generating. We acknowledge that our study needs validation in external and larger cohorts of patients. We excluded from the analysis patients with missing clinical and follow-up data, thus exposing our results to both selection and attrition bias; however, this subgroup was less than 10% of the whole sample size. Moreover, data were from a single-center, thus generalizability is unknown; however, procedures were performed with adherence to local protocols, which allows reproducibility of results.

In summary, refractory occlusion requiring stent placement was associated with ischemic stroke due to atherosclerosis or dissection of the basilar artery and was rare in cardioembolic stroke. Despite good recanalization being obtained in the majority of the study population, in patients with stent placement early neurological improvement was less frequent, whereas periprocedural complications and poor functional outcome were more frequent, reflecting the higher complexity of the endovascular procedure due to timing and stroke etiology. However, stenting in the basilar artery may represent a reasonable therapeutic option and should be considered in those cases of failure of thrombectomy/thrombus aspiration, due to the high burden of mortality and disability of permanent BAO. Our results may inform clinicians about prognosis of stenting as a rescue therapy in BAO, helping communication with patients and their relatives.

## Supplementary Information

Below is the link to the electronic supplementary material.

Supplementary file1 (DOCX 33 KB)

Supplementary file2 (DOCX 126 KB)

## Data Availability

The datasets analyzed during the current study are not publicly available due to patient confidentiality and institutional regulations but can be provided from the corresponding author upon reasonable request and subject to approval by the Ethics Committee.
