# Supplementary material for: Stent placement as rescue treatment in acute basilar artery occlusion
Source: J Neurol. 2026 Jun 8;273(7):376. doi: 10.1007/s00415-026-13922-x (PMC13246889; doi:10.1007/s00415-026-13922-x)
Supplement: Supplementary file 2 — Supplementary file2 (DOCX 126 KB) [file 415_2026_13922_MOESM2_ESM.docx]

**Figure 3 (Supplementary Materials).** Flow diagram of study population.

BA, basilar artery; BAO, basilar artery occlusion; EVT, endovascular treatment; rt-PA, recombinant tissue plasminogen activator.
